# Iron Metabolism in Liver Cancer Stem Cells

**DOI:** 10.3389/fonc.2019.00149

**Published:** 2019-03-19

**Authors:** Stefania Recalcati, Margherita Correnti, Elena Gammella, Chiara Raggi, Pietro Invernizzi, Gaetano Cairo

**Affiliations:** ^1^Department of Biomedical Sciences for Health, University of Milan, Milan, Italy; ^2^Humanitas Clinical and Research Center, IRCCS, Rozzano, Italy; ^3^Department of Experimental and Clinical Medicine, University of Florence, Florence, Italy; ^4^Division of Gastroenterology, Department of Medicine and Surgery, Center for Autoimmune Liver Diseases, San Gerardo Hospital, University of Milano–Bicocca, Monza, Italy

**Keywords:** iron, liver cancer, cancer stem cells, ferroptosis, chelators

## Abstract

Cancer stem cells (CSC) which have been identified in several tumors, including liver cancer, represent a particular subpopulation of tumor cells characterized by properties similar to those of adult stem cells. Importantly, CSC are resistant to standard therapies, thereby leading to metastatic dissemination and tumor relapse. Given the increasing evidence that iron homeostasis is deregulated in cancer, here we describe the iron homeostasis alterations in cancer cells, particularly in liver CSC. We also discuss two paradoxically opposite iron manipulation-strategies for tumor therapy based either on iron chelation or iron overload-mediated oxidant production leading to ferroptosis. A better understanding of iron metabolism modifications occurring in hepatic tumors and particularly in liver CSC cells may offer new therapeutic options for this cancer, which is characterized by increasing incidence and unfavorable prognosis.

## Introduction

The phenotypical and functional heterogeneity of cells within tumors can be explained by both the conventional mechanism centered on clonal evolution and a model based on the presence of cancer stem cells (CSC) which over the last decade received support by increasing experimental evidence. CSC have similar properties to adult stem cells, such as the ability for unlimited self-renewal and differentiation and are believed to be a major source of cancer initiation and progression, thus resulting in a heterogeneous tumor cell progeny (1–3). Moreover, CSC are characterized by drug resistance, an element allowing tumors to survive therapies. In fact, the relatively quiescent CSC can escape cell death after standard chemotherapy treatments, which preferentially eliminate rapidly proliferating cells; as a consequence, the remaining CSC may lead to cancer relapse and metastasis, whose treatment is more complex and often unsatisfactory. Notably, it is now also realized that some of the alterations of iron homeostasis that have recently emerged as key factors in cancer growth and progression are present also in CSC [reviewed in (4)]. In this Review, we discuss current knowledge of the role of iron as a key factor in cancer development, particularly in liver and hepatic CSC, and we also address iron-centered therapeutic approaches.

## Primary Liver Cancer and Cancer Stem Cells

Primary liver cancer (PLC) is the fifth most common cancer worldwide and the second most frequent leading cause of cancer-related mortality and its incidence and mortality are increasingat a fast rate, especially in western countries (5, 6). Hepatocellular carcinoma (HCC) and cholangiocarcinoma (CCA) represent the two major forms of PLC, and account for ~ 90 and 5% of all primary liver tumors, respectively (5, 6).

HCC arises from malignant transformation of hepatocytes and is often associated with known risk factors, such as excessive alcohol intake, infection with hepatitis B virus (HBV) or hepatitis C virus (HCV), aflatoxin B1 intake, fatty infiltration, autoimmune liver diseases and alterations of iron metabolism leading to hepatic iron accumulation like hemochromatosis (5, 7). Unlike the HCV-related HCCs, the incidence of HCC linked to the metabolic syndrome is increasing, principally due to obesity epidemic continuous rising (8).

On the other hand, CCA arise from neoplastic transformation of intra- and extra-hepatic biliary epithelial cells (cholangiocytes) and the frequency of its established risk factors mostly differs depending on geographic area (9). For example, infection with liver flukes (*Opisthorchis viverrini* and *Clonorchis sinensis*) is a common risk factor for CCA development in Southeast Asia (6, 10). Conversely, primary sclerosing cholangitis (PSC) is the most common predisposing condition for CCA in the western countries (6). HBV or HCV infection and cirrhosis have been also proposed as potential etiologies of CCA, shared with HCC (6, 7).

While liver transplantation, surgical resection and locoregional therapies are possible curative options at early phases, the majority of PLC patients unfortunately present advanced stages of the disease, for which treatments are very limited and the prognosis remains unfavorable (5, 11).

Like most other solid tumors, PLC are characterized by an extensive clinical and pathobiological heterogeneity in terms of cellular morphologies as well as genetic and epigenetic landscape (12–14). Such intra-tumor heterogeneity may reflect the presence of different clonal subpopulations exhibiting differential sensitivity to drugs (7, 12, 13). In this respect, the recent advent of the CSC hypothesis has added a new level of complexity in understanding PLC heterogeneity and drug resistance. According to this model, CSC are thought to drive tumor growth and progression, as well as tumor metastasis, recurrence and therapy resistance, representing the unique unit of selection within the tumor (2, 15–17). Interestingly, a new “CSC plasticity model” has been proposed, further increasing the complexity of tumor biology. According to this theory, the different tumor cell subpopulations are highly plastic and dynamic, continuously switching between non-CSC and CSC phenotypes, depending on various intrinsic and extrinsic stimuli (18).

While the CSC existence has been confirmed in HCC (19–22) and recently also in CCA (23, 24), no consensus has yet been reached regarding the origin of hepatic CSC. In addition to the classical idea that CSC originate from normal liver resident stem cells (1), it is now become accepted that CSC may originate also from more committed progenitor cells and mature differentiated tumor cells through a reprogramming process (11, 25, 26). These considerations go hand in hand with the unsettled debate about the true nature of the PLC cell-of-origin, about which a consensus has not been reached, yet.

Another open question concerns the identification of a common recognized method for isolation and subsequent characterization of liver CSC. During the last years several attempts have been made to obtain a cell population enriched in liver CSC using a variety of techniques. The antigenic approach, which is one of the first methods used to isolate CSC, relies on surface CSC markers detection, including CD133, CD44, OV6, CD90, EpCAM, CD13, CD24, CD47 (27). However, the antigenic approach has several shortcomings, such as lack of clearly defined surface markers specific for individual tumor type, such as PLC, and the fact that different cancer cell populations with tumor-initiating activity can be isolated using different markers within a tumor type (18, 28). Therefore, none of the proposed markers is specific for liver CSC and universally expressed in all liver CSC (29). In addition, the surface marker expression can diverge depending on the specific context (28). In addition to the classical antigenic approach, there are several assays based on CSC functional properties, including Side Population (SP) analysis, Aldefluor assay and tumor-sphere formation assay (14, 24, 27). All these functional techniques are based on different key CSC features: the first one on the typical drug resistance of CSC (30, 31), the second one on the measurement of aldehyde dehydrogenase activity (32) and the last one on long-term self-renewal capability of CSC (21, 24, 33). Possibly, a combinatorial strategy might be a valid alternative to isolate a better-defined PLC stem-like subset, but the gold-standard approach to evaluate CSC tumorigenic potential remains the *in vivo* approach based on xenotransplantation in immune-deficient mice (14, 24, 27).

## Iron and Liver Cancer

**Figure 1:**
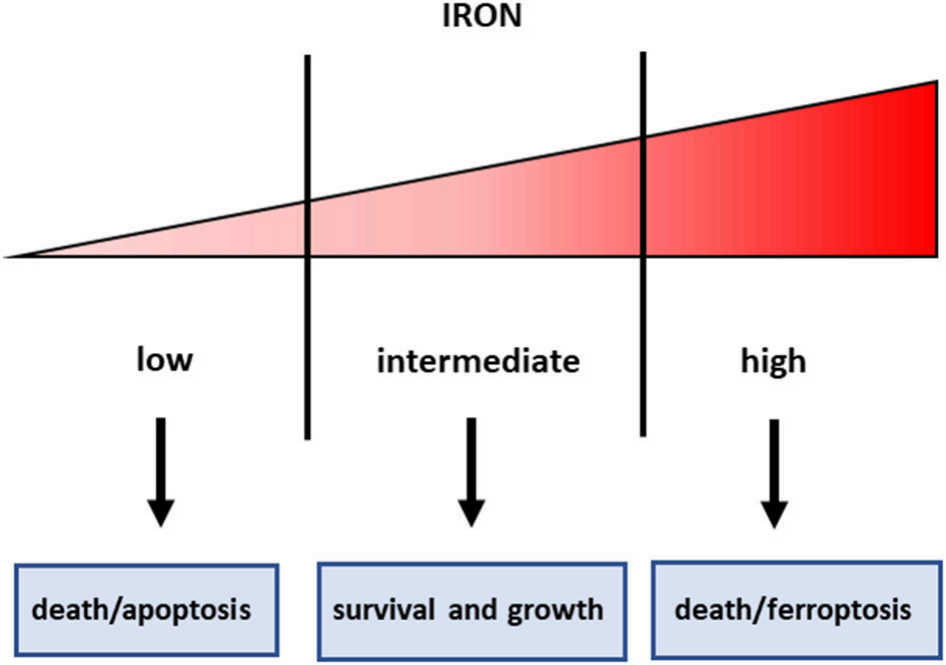
Iron threshold concept. Certain iron levels are required for cell survival and homeostasis, but iron concentrations too low lead to apoptotic cell death, whereas excess iron equally triggers cell death.

**Figure 2:**
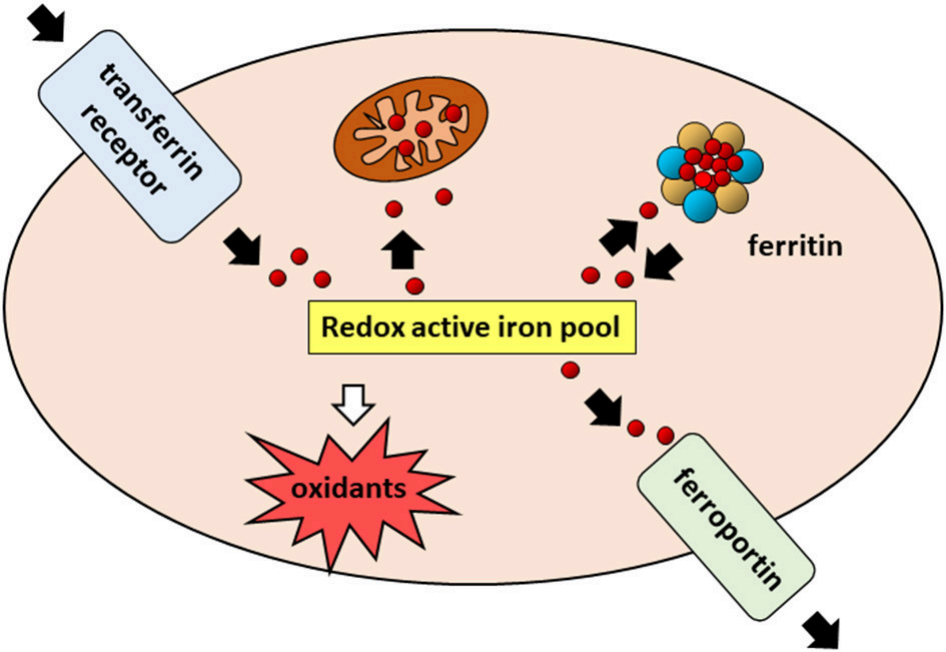
Cellular iron pathways in a nutshell. Transferrin bound iron, internalized through endocytosis of the transferrin receptor (TfR1), enters a pool of redox-active iron whose concentration is kept under control by mechanisms ensuring that the iron which is not used for biochemical processes, particularly in mitochondria, is either safely stored in cytoplasmic ferritin or exported by ferroportin.

Iron is an essential component of living organisms, as it is necessary for cellular metabolism, replication and growth. However, excess iron can facilitate the generation of the most reactive and toxic forms of oxidants through the Fenton reaction (34); therefore, iron levels are carefully kept within an optimal range at both systemic and cellular levels (Figure 1). The major players in maintaining cellular iron homeostasis are the transferrin receptor (TfR1) that internalizes transferrin-bound iron, ferroportin (Fpn), the only cellular iron exporter, and ferritin that stores excess iron (35) (Figure 2). A number of epidemiological studies indicate a positive association between cancer and high body iron content in the general population (36). Since the liver is the organ where excess iron accumulates (37) and plays an important role in maintaining iron homeostasis, a large body of evidence from human, animal, and *in vitro* studies supports the positive relation between increased body iron stores and the risk of liver cancer. In fact, HCC is the prevalent tumor found in hemochromatosis patients (38).

Studies investigating cancer risk in subjects undergoing blood transfusion or phlebotomy suggest that iron excess is not merely associated with cancer but plays an active role in carcinogenesis. The biological basis of the association between iron and cancer is double-face as it probably rests in both oxidative stress-mediated DNA damage and availability of the metal to support fast growth (39). Iron may therefore play a role both as an initiator in an early phase and, once malignant change has occurred, as a promoter that allows the transformed cell to fully express its potential of unrestricted growth.

In addition, recent studies showed that both systemic and cellular iron metabolism is altered in tumors (40). In general, given the high iron needs of tumor cells to sustain cell proliferation, the alterations of iron trafficking in cancer cells lead to iron acquisition. To this purpose, cancer cells usually increase iron uptake, for example by up-regulating TfR1, decrease iron release by inhibiting Fpn, or both. Several studies have demonstrated that these alterations of cellular iron metabolism are directly dependent on the action of oncogenes and tumor suppressors (39). Notably, the “iron addiction” of tumors was confirmed by the analysis of different cell lines using a novel method (41), which showed that cancer cells had significantly increased redox-active iron pools compared to non-tumorigenic cells. The role of iron in cancer is not related only to the “iron seeking” phenotype of most cancer cells. In fact, iron levels can modulate apoptosis in multiple ways, for example by affecting the alternative splicing of Fas/CD95 transcripts between the pro-apoptotic and anti-apoptotic isoforms (42). Moreover, the p53 pathway that regulates cell cycle and apoptosis interacts with iron metabolism in a complicated crosstalk that remains to be completely explained (43). As an example of opposing observations regarding the involvement of iron and p53 in the pathogenesis of HCC, it has been shown that exposure to iron down-regulated MDM2, the ubiquitin ligase which leads to degradation of p53 (44), whereas another study found decreased p53 protein levels in the liver of iron overloaded mice (45).

Iron metabolism has been investigated in rodent models of hepatic carcinogenesis as well as in regenerating liver, which represents an excellent example of controlled liver proliferation and hence a powerful model system to get insights into the processes leading to hepatocarcinogenesis. Similarly to other types of growing cells, increased expression of TfR1 has been found in rat liver preneoplastic nodules and HCC (46, 47), as well as in regenerating liver cells (48), probably in order to insure sufficient iron to sustain cell proliferation. Recently, a study investigating iron metabolism gene expression and prognostic features of HCC found that TfR1 are more expressed in HCC than in surrounding liver and correlate with poorer prognosis (49). Researches also addressed the role of iron in CCA, a severe liver tumor with limited therapeutic possibilities, concluding that high expression of TfR1, with consequent iron uptake, contributes to CCA progression and poorer clinical outcomes (50). Accordingly, we showed that elevated iron content is a negative prognostic factor in CCA patients (51).

However, recent studies indicated that altered expression of proteins of iron metabolism like TfR1 in tumor cells is not only a system to acquire more iron but may impinge on tumor growth in an iron-independent manner. In fact, in line with the demonstrated interaction of TfR1 with ligands other than transferrin (Tf) (52), it appears that, in addition to its role in iron uptake, TfR1 activates signaling pathways and has a role in apoptosis, a key process in cancer development. For example, TfR1, by activating JNK upon phosphorylation by Src, impairs apoptosis and thereby increases breast cancer cell survival (53). In addition, the interaction of TfR1 with Tf may also have roles which are different from iron uptake but are still important for tumor cell growth; in fact TfR1 appears to be implicated in epithelial mesenchymal transition (EMT) (54), which is an important process for cancer progression, and metastatic growth (55).

Over the last years, a number of studies have shown that the levels of Fpn are reduced in several cancer cells compared to their nonmalignant counterparts, so that Fpn downregulation appears as a common strategy that a variety of cancer cells adopt to enhance intracellular iron availability (39). Interestingly, Fpn expression is a strong and independent predictor of prognosis in different tumor types (39). Dysregulation of the hepcidin/Fpn axis may also play a relevant role in liver tumors. Recently, it has been shown that hypermethylation of specific sequences in the promoter region of the gene coding for hepcidin, the liver hormone that regulates iron homeostasis by inhibiting Fpn-mediated iron export, results in transcriptional downregulation of hepcidin expression in HCC (56). A similar effect was found in a model of rat liver carcinogenesis, in which the downregulation of hepcidin and the consequent increase of Fpn-mediated iron release may underlie the decreased intracellular levels of iron in preneoplastic foci (57). This could be a specific feature of hepatic cancer, as Fpn is usually repressed in cancer cells and low iron levels are not attributed to increased iron export but to higher consumption. However, we found significantly reduced Fpn mRNA levels in tumor samples from CCA patients compared to matched surrounding liver, suggesting that also in PLC elevated iron content is a negative prognostic factor (51).

The reprogramming of iron metabolism in tumors comprises the repression of the iron storage protein ferritin, as a mean to increase iron availability for the high requirements of cancer cell (58). Conversely, a tumor-suppressive role for ferritin has been shown in several types of cancer, such as breast and colorectal cancer. Ferritin expression appears to be directly modulated by both oncogenes, which down-regulate ferritin, and tumor suppressors, which induce ferritin expression [reviewed in Torti (39)]. Accordingly, a recent study highlighted a new mechanism based on a complex miRNA network by which oncogenic miRNAs inhibit the expression of H ferritin and its pseudogenes, thus leading to prostate cancer growth (59). Ferritin expression in liver cancer has been investigated by a number of studies in rodent models of hepatocarcinogenesis and in human hepatomas (60), but a coherent picture has not emerged, probably as the result of different experimental approaches and models, but also because of the multiple mechanisms of regulation of this protein, which is affected by iron status, differentiation, growth rate, inflammation, etc. (61). Therefore, whether increased ferritin levels in HCC patients merely reflect increased accumulation of iron, which is the actual carcinogen, or play an active role in malignant transformation is still unknown.

## Liver CSC and Iron

Over the last years, several investigations found increased iron content in CSC of several types of tumors and also showed that altered iron trafficking is functional to the role of CSC in tumor growth. Indeed, variations of iron levels in CSC were achieved by specific modulation of the expression of genes of iron metabolism; in particular, TfR1-dependent iron uptake is induced whereas Fpn-mediated iron export is down-modulated. In most studies, the enhanced iron content was mirrored by high levels of the iron storage protein ferritin (4, 62).

Support to the idea that higher iron levels have a functional role in CSC formation and the maintenance of stemness was provided by evidence that iron chelation inhibited tumor spheres (a proxy of CSC) formation in several types of cancers (4). Additionally, manipulation of iron levels modulated the expression of typical stemness markers (4). Of course, it is also conceivable that alterations of iron homeostasis induced by genes related to CSC, such as Myc-mediated inhibition of ferritin expression (63), cooperate in order to disrupt iron homeostasis in CSC.

Notably, *in vivo* studies showing higher tumorigenic capacity of iron-rich tumor spheres in mouse xenograft tumor models confirmed the role of iron (64–66). Moreover, poorer prognosis for human tumors with altered expression of iron proteins in CSC is a common finding (51, 64, 66–68).

As it regards the role of iron in liver CSC, knowledge is limited. We recently showed that the regulation of iron homeostasis is profoundly different if CCA cell lines are cultured under conditions inducing the formation of tumor spheres, as compared to CCA cells growing in monolayers. In particular, we found high H ferritin levels and TfR1 expression accompanied by diminished Fpn transcription, a pattern leading to elevated iron content. Moreover, this finding was mirrored by data showing a trend toward shorter survival in CCA patients with high expression of ferritin and hepcidin (51).

## Iron and Cancer Therapy

In consideration of the role of iron in cancer, and particularly in CSC that are resistant to radio and chemotherapy, manipulation of iron levels appears a promising therapeutic strategy. Given the double-edged sword property of iron in controlling cell fate (Figure 1), both iron chelation (in order to starve tumor cells for this essential micronutrient) and iron overload (in order to exploit iron toxicity) have been proposed for cancer treatment, but there are still several concerns for the use of both strategies. In fact, the threshold above which iron levels are no longer supportive of growth but become toxic is not well-defined, even though recent findings indicated that the functional iron concentration that allows *in vitro* cell proliferation is very low (i.e., in the nanomolar range) (69). Iron chelation has been used in several types of cancer, including HCC (70). However, since desferrioxamine, an iron chelator in use since the nineties, has a rather poor bioavailability, limited success in treating cancer has been obtained with iron chelators so far, though more recently developed oral iron chelators like deferasirox showed some effect in leukemia patients (71). The major mechanisms by which sequestration of intracellular iron by classical iron chelators targets tumor cells are:(i) inhibition of the iron-containing ribonucleotide reductase, the rate-limiting enzyme for DNA synthesis, (ii) cyclin dependent kinases-mediated induction of cell cycle arrest, iii) activation of metastasis and tumor suppressor genes, such as NDRG1 and p53, respectively. Moreover, recent data indicate that chelators can also suppress cancer by inhibiting the EMT, a key characteristic of CSC (72). Since the prolyl hydroxylases controlling the levels of the hypoxia inducible factors (HIF) are iron dependent enzymes, iron chelators induce HIF and its numerous target genes (73). We have shown that HIF-1 is involved in the protective effect exerted by the iron chelator dexrazoxane against anthracycline cardiotoxicity (74). Therefore, one may legitimately wonder whether iron chelators may have a similar effect in cancer cells, thus limiting the therapeutic effect of anticancer drugs.

A new class of iron chelators, such as the thiosemicarbazone Dp44m, which were reported to inhibit the proliferation of cancer cell lines *in vitro* by inducing the expression of p21, a cyclin-dependent kinase inhibitor involved in cell cycle arrest (75), appear promising. Opposite to conventional iron chelators like desferrioxamine, Dp44m is a tridentate ligand that forms redox active iron complexes leading to increased oxidant levels and cytotoxicity (76). These compounds also limit the growth of tumor xenografts in nude mice and have entered clinical trials, but their effect on CSC has not been tested. However, considering the well-known drug resistance of CSC, it is worth to mention that lysosomal-targeted Dp44m prevents the sequestration of a chemotherapeutic anthracycline like doxorubicin in lysosomes, which is triggered by the stressful environment of the tumor (77). Through this mechanism, which allows doxorubicin to exert its toxic effects in the nucleus and the mitochondria, this chelator may thus favor the action of anticancer drugs. In the same line, a recent study showed that a novel iron chelator, DpdtC (di-2-pyridylketone hydrazone dithiocarbamate) can induce lysosomal oxidant production and growth inhibition of HCC cell lines (78).

The use of these compounds represents therefore an approach similar to that relying on the toxic side of iron for killing cancer cells (see below). This strategy gained momentum after the discovery of ferroptosis, a form on non-apoptotic cell death caused by iron-dependent production and accumulation of reactive and toxic hydroperoxides (79). Iron plays a dual role in ferroptosis, as iron on the one hand can promote Fenton chemistry and on the other hand stimulate the activity of the iron-dependent enzyme lipoxygenases that contribute to ferroptosis by degrading polyunsaturated fatty-acid-containing phospholipids (80). Malignant cells generate high levels of oxidants as by-products of the biosynthesis of macromolecules and must balance iron uptake for proliferation with the risk of generating oxidative stress (81). Since most ferroptosis-inducing agents have limited use *in vivo* due to low bioavailability (82), iron may thus have potential to trigger ferroptotic cell death in cancer, as high iron concentrations may push malignant cells beyond the breaking point of oxidative stress tolerance (34). Interestingly, it has been shown that iron is required to induce ferroptosis also in drug-resistant “persister” cancer cells, thus showing therapeutic promise to eliminate this pool of tumor cells characterized by non-mutational drug-tolerance (83).

Alternatively, iron may potentiate the effect of anticancer drugs like the multikinase inhibitor sorafenib, which is used for treating HCC. In fact, the evidence that iron chelation protected HCC cells against iron-dependent oxidative stress caused by sorafenib indicates that ferroptosis can represent an inhibitory mechanism of the growth of liver cancer cells (84). A further indication of the role of iron and ferroptosis in PLC was provided by a study showing that ferritin induction protects HCC cells from ferroptosis (85).

Triggering ferroptosis appears a promising approach also to attack CSC, which represent a negative factor for cancer outcome (86). Ovarian CSC showed higher sensitivity to ferroptosis than non-tumorigenic ovarian stem cells (66) and a recent study showed that in breast CSC exposure to salinomycin and its derivatives leads to lysosomal iron accumulation, oxidants production and ferroptosis (87). Similarly, increased intracellular iron levels provided by ferritin degradation can lead glioblastoma cells to ferroptosis (64). However, a number of recent studies have shown that CSC are iron-rich and iron-dependent (4); therefore, enhancing cellular iron may not always be an effective strategy to eliminate CSC selectively. Moreover, in general, low levels of oxidants have been reported in CSC, making difficult the approach based on targeting iron-dependent, oxidant related pathways in CSC. In fact, we found that sphere-forming CCA cells, in spite of higher levels of oxidants and iron, were more resistant to the ferroptosis inducer erastin than their counterpart growing as monolayer (51). It seems therefore that, before recommending the manipulation of iron homeostasis as a therapeutic tool for targeting tumors and the use of iron supplementation to promote ferroptosis of cancer cells, additional studies are needed to understand the role of iron in the pathways controlling cell death, as iron can possibly promote CSC cell growth, thereby affecting the survival of cancer patients.

It should be also kept in mind that HCC almost always develop in the context of chronic liver disease characterized by persistent damage and inflammation, which can be further stimulated by iron supplementation.

## Targeting Iron to Cancer Cells

Administration of massive quantities of iron successfully killed multiple myeloma cells (88), though these cells, which secrete large amounts of disulfide-rich immunoglobulins and are thus a source of oxidants (89), may be particularly sensitive to iron-dependent oxidative stress. Iron may also mediate the effect of high i.v. doses of vitamin C that were reported to kill liver CSC specifically both *in vitro* and *in vivo* by promoting oxidant production (90). This paradoxical effect of a recognized antioxidant may be explained by the strong reducing properties of ascorbic acid which, at pharmacological concentrations (>1 mM), maintains iron in the highly reactive ferrous form, thereby increasing, instead of preventing, oxidative stress and cell death.

How to load cancer cells, in particular CSC, with iron *in vivo*, possibly in a specific way ? Oral iron is poorly adsorbed and its uptake is subjected to a strict feed-back regulation (91), therefore, even recurring to novel nanoparticulate ferritin core mimetics (92), it does not appear a promising approach. Parenteral iron preparations, such as dextran iron, have higher efficacy but relatively poor safety due to hypersensitivity reactions. However, new formulations, such as iron gluconate and iron sucrose, do not present toxicity issues and two iv iron compounds prepared with new pharmaceutical technologies are currently approved for the treatment of iron deficiency anemia (93) and could be used in cancer patients. These iron complexes are endocytosed and processed by macrophages within the reticuloendothelial system, mainly in the liver, spleen and bone marrow, but the precise mechanism of recognition and internalization is not fully defined (94). Inside the macrophages, iron is released from the iron–carbohydrate complex in acidic endo-lysosomes through a mechanism incompletely understood and subsequently transported to the cytoplasm, where it can be stored in ferritin or exported into the bloodstream by Fpn.

An approach alternative to exogenous iron administration is to impair safe intracellular iron storage, for example by triggering lysosomal ferritin degradation. Indeed, treatment of breast CSC with salinomycin resulted in increased ferritin degradation in lysosomes; the iron released then facilitated oxidants production and ferroptosis (87). An analogous release of catalytic ferrous iron from ferritin led mesothelioma cells to death after exposure to non-thermal plasma, which produces hydroxyl radicals (95). Similarly, artesunate, by enhancing lysosomal ferritin degradation, was able to induce cell death in HCC cell lines (96). Notably, regulated autophagic degradation of ferritin (ferritinophagy) contributes to ferroptosis and was found to occur in primary human hepatic stellate cells obtained from liver tissue of advanced fibrotic patients with HCC, thereby alleviating liver fibrosis (97). However, ferritin is not only, or not always, a source of iron for Fenton chemistry. Iron storage inside ferritin is a protective stratagem against iron-mediated oxidative injury (34, 61) and also mitochondrial ferritin shields this important organelle from oxidants damage (98). The relevance of this function in cancer has been shown by a study reporting that high ferritin expression in myeloma cells is directly related to increased resistance to oxidants generated by exposure to the proteasome inhibitor bortezomib (88). A similar effect was found in HCC cells in which oxidative stress mediated induction of ferritin protected from ferroptosis (85).

The alterations of iron homeostasis seem to involve not only cancer cells, but also other cell types of the tumor microenvironment, particularly macrophages. In response to signals in the tumor microenvironment, tumor-associated macrophages (TAM), which favor tumor growth and progression, often become similar to M2 polarized macrophages endowed with anti-inflammatory activity, which display a gene expression profile characterized by active iron uptake and release and ferritin repression (99, 100). Therefore, this kind of iron metabolism in TAM macrophages might promote tumor growth by providing iron to adjacent tumor cells (101). Notably, also CCA CSC prime TAM toward a tumor-promoting phenotype, although iron metabolism has not been explored in this setting (24). However, it should be noted that the TAM population may be heterogeneous (102), as it has been found that in one type of murine prostate cancer, but not in another model of prostate cancer, some TAM contain iron aggregates typical of iron storing macrophages (103, 104) and in ovarian cancer TAM presented a prevalence of M1 phenotype (105). In this case, TAM, by sequestering iron, may limit its availability to cancer cells, thus impairing tumor growth. On the other hand, accumulation of an excess of exogenously administered iron, in the same way as an excess of heme iron in hemorrhagic tumor regions (106), may induce a switch of TAM toward the M1 antitumor phenotype, which is associated with the most favorable prognosis, as recently confirmed by extensive immunogenomic analysis of thousands of diverse tumor types (102).

It should be noted that in malignancies induced (or accompanied) by constant damage and chronic inflammation like HCC, two factors can further impinge on iron trafficking: on the one hand, TAM are more M1-like (107) and could restrict iron availability in the microenvironment and exert toxicity against malignant cells; on the other hand, the high hepcidin levels caused by inflammation may weaken Fpn-mediated iron release from macrophages, thus contrasting the iron-donating activity of TAM. These considerations are at odd with the correlation between high hepcidin levels and tumor progression in breast cancer patients (108, 109) and poorer prognosis in CCA (51). However, hepcidin may interact with Fpn expressed by both TAM and cancer cells; moreover, other iron transporters like lipocalin2 (110) may be involved.

While the role of iron in TAM, which seems clearly context-dependent, remains to be fully clarified, iron handling by TAM may have therapeutic implications. In fact, a seemingly promising approach relies on the use of iron oxide nanoparticles, a type of nanocarriers used for cancer targeted drug delivery, which are internalized by macrophages, including TAM. In line with *in vitro* data showing that superparamagnetic iron oxides induce a macrophage shift from the M2 to the M1 subtype (111), a recent study showed that iron oxide nanoparticles inhibited tumor growth indirectly by inducing M1 polarization. Highly increased iron levels in TAM resulted in oxidants production and cancer cell apoptosis (112). While the specific targeting of some species of drug delivering nanoparticles to tumors relies on the higher permeability of leaky blood vessels inside the cancerous mass, thanks to the magnetic properties of iron oxide nanoparticles, a localized external magnetic field can be used to guide these nanoparticles to tumors, thus achieving an improved therapeutic response and reducing side effects.

## Conclusions

There is growing evidence that iron homeostasis is dysregulated in cancer, including PLC, and over the past few years also insights into the key role of iron in CSC have emerged. CSC show alterations of iron metabolism leading to a phenotype characterized by elevated cellular iron content, so that the expression of their typical features, such as stemness, is inhibited by iron chelators, thus suggesting the use of these compounds for CSC-targeted therapy. On the other hand, most recent therapeutic approaches seem aimed at exploiting the capacity of excess iron to induce ferroptotic cell death in cancer cells. However, given the involvement of iron in many important pathophysiological settings, it should be considered that we need to better understand how manipulation of iron levels to contrast tumor growth may interfere with iron homeostasis in healthy tissues or worsen conditions accompanying cancer, such as inflammation or anemia. Moreover, unfortunately, the mechanism(s) underlying the redox regulation in CSC are still not fully understood, as indicated by the higher resistance of CCA CSC to ferroptosis despite a higher basal oxidative stress condition (51).

Despite the still limited understanding of many processes, the increasing recognition of the importance of iron in cancer biology offers new chances to unravel tumor pathogenesis and thus develop more effective iron-centered therapeutic strategies against liver cancer.

## Author Contributions

MC, SR, and GC wrote the manuscript. CR, EG, and PI made significant revisions to the manuscript. All authors read and approved the final manuscript.

### Conflict of Interest Statement

The authors declare that the research was conducted in the absence of any commercial or financial relationships that could be construed as a potential conflict of interest.
